# Monte Carlo modeling of HD120 multileaf collimator on Varian TrueBeam linear accelerator for verification of 6X and 6X FFF VMAT SABR treatment plans

**DOI:** 10.1120/jacmp.v15i3.4686

**Published:** 2014-05-08

**Authors:** Alanah M. Bergman, Ermias Gete, Cheryl Duzenli, Tony Teke

**Affiliations:** ^1^ Medical Physics BC Cancer Agency ‐ Vancouver Centre Vancouver BC; ^2^ Medical Physics BC Cancer Agency ‐ Centre for Southern Interior Kelowna BC Canada

**Keywords:** flattening filter‐free, Monte Carlo, VMAT, SABR, SBRT

## Abstract

A Monte Carlo (MC) validation of the vendor‐supplied Varian TrueBeam 6 MV flattened (6X) phase‐space file and the first implementation of the Siebers‐Keall MC MLC model as applied to the HD120 MLC (for 6X flat and 6X flattening filterfree (6X FFF) beams) are described. The MC model is validated in the context of VMAT patient‐specific quality assurance. The Monte Carlo commissioning process involves: 1) validating the calculated open‐field percentage depth doses (PDDs), profiles, and output factors (OF), 2) adapting the Siebers‐Keall MLC model to match the new HD120‐MLC geometry and material composition, 3) determining the absolute dose conversion factor for the MC calculation, and 4) validating this entire linac/MLC in the context of dose calculation verification for clinical VMAT plans. MC PDDs for the 6X beams agree with the measured data to within 2.0% for field sizes ranging from 2 × 2 to 40 × 40 cm^2^. Measured and MC profiles show agreement in the 50% field width and the 80%‐20% penumbra region to within 1.3 mm for all square field sizes. MC OFs for the 2 to 40 cm^2^ square fields agree with measurement to within 1.6%. Verification of VMAT SABR lung, liver, and vertebra plans demonstrate that measured and MC ion chamber doses agree within 0.6% for the 6X beam and within 2.0% for the 6X FFF beam. A 3D gamma factor analysis demonstrates that for the 6X beam, > 99% of voxels meet the pass criteria (3%/3 mm). For the 6X FFF beam, > 94% of voxels meet this criteria. The TrueBeam accelerator delivering 6X and 6X FFF beams with the HD120 MLC can be modeled in Monte Carlo to provide an independent 3D dose calculation for clinical VMAT plans. This quality assurance tool has been used clinically to verify over 140 6X and 16 6X FFF TrueBeam treatment plans.

PACS number: 87.55.K‐

## I. INTRODUCTION

Volumetric‐modulated arc therapy (VMAT) is a popular treatment option for both radical radiation therapy and stereotactic ablative radiotherapy (SABR) treatments.^(1)^ Treatment sites such as lung, liver, pancreas, adrenal gland, and spine have been recently reported for VMAT SABR.^2^, ^3^, ^4^, ^5^ VMAT is a radiation arc delivery technique with time‐dependent MLC shapes, gantry speed, and dose rates.^6^, ^7^, ^8^ These complex treatments require patient‐specific dose verification, in addition to a rigorous VMAT‐specific, machine quality assurance program.^(9)^ It has been demonstrated that Monte Carlo (MC) simulations are a viable method for validating VMAT plans.^10^, ^11^, ^12^, ^13^, ^14^, ^15^, ^16^, ^17^, ^18^, ^19^ For the Varian iX linear accelerator equipped with the Millennium multileaf collimator (min leaf width=0.5 cm), the MC verification of 6 MV flattened (6X) VMAT treatment plans has been reported.^10^, ^16^, ^17^, ^18^

The TrueBeam linear accelerator is the most recent offering from Varian. According to the company, the head design has been completely reengineered compared to previous models. General TrueBeam commissioning characteristics and comparisons to other Varian linacs are available in the literature.^20^, ^21^ The MC verification of VMAT plans for the 6X FFF beams from the TrueBeam equipped with a Millennium MLC has been reported.^(12)^ For the HD120‐leaf MLC (min leaf width=0.25 cm), a new set of geometric and dosimetric parameters must be determined for the Monte Carlo model. For the TrueBeam linear accelerator, a full MC simulation of the treatment head components (target, primary collimator, flattening filter, monitor chamber) is not possible as the information is proprietary. Instead, precalculated phase spaces located above the secondary collimator (jaws) are provided by the vendor. The 6X phase space was generated using the GEANT4 MC code and validated by Constantin et al.^(22)^ Varian has made available this 6X, as well as the 6X FFF, phase‐space data (other energies also available). The TrueBeam 6X FFF phase space has been independently verified by Gete et al.^(12)^ The 6X beam is independently verified in this manuscript for commissioning of a clinical quality assurance tool.

The TrueBeam STx linac is equipped with a ‘high definition’ 120‐leaf multileaf collimator (HD120 MLC) which features two banks of 60 tungsten leaves. The central 8 cm is comprised of 32×0.25 cm wide leaves (projected at isocenter). The outer 14 cm is comprised of 28×0.50 cm wide leaves. The maximum MLC‐defined field length perpendicular to leaf motion is 22.0 cm at 100 cm from the X‐ray source.

There are several different approaches to modeling the MLC in a Monte Carlo context. The AAPM TG‐105 report^(23)^ divides the different types of models into three categories: a) pseudoexplicit transport (simplified MLC model to modify a fluence map/intensity distribution), b) explicit transport (direct simulation of all particle transport through MLC), and c) explicit‐approximate transport (particle transport, but with some simplifying and speed‐enhancing assumptions). For static‐field IMRT applications, all three of the types of models described in TG‐105 have been employed.^11^, ^24^, ^25^, ^26^, ^27^, ^28^, ^29^, ^30^, ^31^, ^32^, ^33^, ^34^, ^35^

Fix et al.^(36)^ have reported on explicit Monte Carlo simulations of the Varian HD120 using the VMC++ simulation engine. The HD120 MLC was validated for 6 and 15 MV flattened beams generated by a Varian Novalis TX accelerator. Borges et al.^(37)^ adapted the Heath ‘DYNVMLC’ component module to work with the Varian HD120‐leaf MLC^30^. The validation of this model was performed using a 6 MV flattened beam from a Varian Trilogy (2300C/D) machine. Finally, Vazquez‐Quino et al.^(38)^ also modeled the HD120 using the BEAMnrc component VARMLC for a Varian Novalis Tx accelerator 6X flat beam.

In this manuscript, an explicit‐approximate method developed by Keall et al.^(33)^ and Siebers et al.^(34)^ is modified for use with the Varian HD120 MLC. This is a particle transport method that is simplified compared to a fully explicit transport method by making some reasonable assumptions. First, only first scattered Compton photons within the MLC are transported. Second, all electron interactions, photoelectric effect, pair production, and higher order Compton scatter interactions within the MLC material are ignored. Geometric considerations such as intraleaf thickness variation, interleaf leakage, leaf tip thickness, tongue‐and‐groove effects, and leaf‐edge effects are taken into account. Schmidhalter et al.^(39)^ reported on the impact of using different simplifying assumptions in terms of accuracy and calculation efficiency. This particular MLC particle transport model (which will be referred to as the particleDMLC code) has been adapted for the HD120 MLC and implemented clinically to allow for MC quality assurance simulations through the Varian HD120 multileaf collimator.

It should be noted that, unless the MLC leaf motion is synchronized with a monitor unit (MU) index or gantry position, none of the stand‐alone MLC models mentioned above can be used for a VMAT single‐run Monte Carlo plan simulation/verification. The particleDMLC code implemented here was modified to provide this synchronization using the method described by Lobo and Popescu.^(15)^ This group has since generalized the concept to provide synchronization capability in the component modules SYNCVMLC (for the Millennium MLC, available as part of the 2011 release of BEAMnrc) and SYNCHDMLC.^(40)^

The particleDMLC model, combined with a validated TrueBeam 6X and 6X FFF open‐beam phase space and a time‐dependent beam configuration source model, allows for the accurate simulation/verification of VMAT treatment plans in Monte Carlo. To our knowledge, a MC model of the HD120 MLC has not been validated in the context of a Varian TrueBeam 6X FFF beam, particularly for VMAT‐type applications.

## II. MATERIALS AND METHODS

### A. Monte Carlo simulations

#### A.1 Varian TrueBeam phase space

The detailed specifications of the components of the TrueBeam accelerator head (Varian Medical Systems, Palo Alto, CA) are proprietary and not available to the public for direct simulations. For Monte Carlo users, Varian provides IAEA‐compliant phase‐space files located just above the secondary X/Y collimator. Constantin et al.^(22)^ reported on the generation and validation of the 6 MV (flattened) open‐field phase space. Simulations through the linac head were performed with the GEANT4 MC code. The phase space was scored onto a surface of a cylinder located above the secondary collimator. In its current form, this phase space cannot be adjusted or ‘tweaked’ to match a particular linear accelerator (i.e., cannot change the electron beam energy and/or spot size/shape on target) and must be accepted 'as is'. The usual steps involved in validating the MC model of the accelerator can be undertaken using this phase space as a starting point. The 6X phase‐space file used here contains $85\times 10^{6}$ particles. The 6X FFF phase‐space file contains $74\times 10^{6}$ particles.

The curved phase space from the GEANT4 simulations are not compatible with the BEAMnrc package. A method to convert this phase‐space file into a format compatible with BEAMnrc for benchmarking has been reported for the 6X FFF energy mode.^(12)^ The modified 6X planar phase space is transported through the secondary collimator (X/Y jaws) for a specified field size. A second (now jaw size‐specific) phase space is stored at 55.0 cm from the target for repeated input into the open field or VMAT simulations in the voxelized patient or verification phantom.

#### A.2 BEAMnrc source model

The simulations in this manuscript were performed with the BEAMnrc/DOSXYZnrc Monte Carlo code.^(41)^ Dose to the patient/phantom is simulated using ‘Source 20’ developed by Lobo and Popescu^(15)^ for the 2011 release of BEAMnrc/DOSXYZnrc. This source does allow for time‐dependent beam configurations (gantry, collimator, MLC, couch position, dose rate, etc.) starting from a phase space located below the secondary collimation which lends itself very well to both static‐field and VMAT‐type applications (multiple 360° arcs can be simulated in a single run). The DOSXYZnrc source will call for particles dynamically from BEAMnrc and any MLC modeling code via the use of shared libraries. Generally, for the BEAMnrc and DOSXYZnrc simulations, the number of histories is chosen to achieve an overall statistical uncertainty in the high‐dose region of ≤2.0%.

#### A.3 6 MV (flattened) percentage depth doses (PDD)/profiles

DOSXYZnrc is used to call jaw size‐specific phase‐space file data for simulation of open‐field percentage depth doses (PDD) and profiles and in a voxelized phantom (SSD 100 cm). PDDs for jaw‐defined field sizes of 2×2, 3×3, 5×5, 10×10, 20×20, and $40\times 40\;{\text{cm}}^{2}$ are simulated. MC PDD calculations are performed on a cubic, water‐equivalent phantom (voxel size=5 mm (in‐plane)×3 mm (cross‐plane)×5 mm (depth)). The number of histories run in DOSXYZnrc is 500 million for field sizes $<10\times 10\;{\text{cm}}^{2}$ and 900 million for field sizes $>10\times 10\;{\text{cm}}^{2}$. This is required to achieve a statistical dose error of <1% on the Monte Carlo simulations. MC runs use the following parameters: 1) AP=PCUT=0.010 MeV, and 2) AE=ECUT=0.700 MeV Photon/electron splitting is invoked with a split factor of 20.

The cross‐plane profiles at a depth of 5.0 cm are also simulated using MC. The voxel size for the MC simulations for field sizes $\leq 10\times 10\;{\text{cm}}^{2}$ is $5\times 1\;(\text{cross}‐\text{plane})\times 5\;{\text{mm}}^{3}$ and for field sizes $>10\times 10\;{\text{cm}}^{2}$, $5\times 3\times 5\;{\text{mm}}^{3}$.

Percentage depth‐dose and cross‐plane profiles are measured in a water tank using a CC13 (0.13 cc/cavity radius=3 mm) chamber (IBA Dosimetry, Schwarzenbruck, Germany) for field sizes $>10\times 10\;{\text{cm}}^{2}$ and an IBA CC01 (0.01 cc/cavity radius=1 mm) chamber for field sizes $\leq 10\times 10\;{\text{cm}}^{2}$. Measurements were repeated using an electron field diode (EFD) (IBA Dosimetry) for the small field sizes, and no appreciable difference in PDD/profile scans compared to the CC01 chamber were noted.

#### A.4 Output factors

Output factors (relative to d=10 cm, $\text{field size}=10\times 10\;{\text{cm}}^{2}$) for square fields sizes ranging from 2×2 to $40\times 40\;{\text{cm}}^{2}$ are measured in water with an ion chamber and compared to MC calculation. Asymmetric field sizes (4×40 and $40\times 4\;{\text{cm}}^{2}$) are also assessed. Output factors are measured in a water tank using the CC13 chamber for field sizes $>10\times 10\;{\text{cm}}^{2}$ and the CC01 chamber for field sizes $\leq 10\times 10\;{\text{cm}}^{2}$. Measurements made in the $2\times 2\;{\text{cm}}^{2}$ field with the small (0.01 cc) volume chamber are renormalized using the daisy‐chain method described by Dieterich and Sherouse^(42)^ and adopted by Gete et al.^(12)^ The ‘intermediate’ field size that is used in the normalization is the $3\times 3\;{\text{cm}}^{2}$ field, consistent with the Gete implementation.

The phantom for MC simulations has a nonuniform voxel size of $5\times 5\times 10\;{\text{mm}}^{3}$ (crossplane×in‐plane×depth). For the calculation of output factors, the following MC transport parameters were used: AP=PCUT=0.010 MeV, AE=ECUT=0.521 MeV, and photon/electron splitting factor of 20. The EXACT boundary crossing algorithm was used with the nonuniform voxel size Monte Carlo phantom.

#### A.5 Postprocessing: absolute dose conversion

Dose distributions from DOSXYZnrc can be converted from MC dose/particle incident‐on‐target to absolute dose using a virtual linac calibration method that fully accounts for the changes in backscatter from the secondary collimator to the monitor chamber of any given treatment field.^(43)^ Having this information about the monitor chamber design is important for some models of accelerator (e.g., Varian iX), as the backscatter contribution can change with varying X/Y collimator settings (particularly for very asymmetric fields).^44^, ^45^ For the TrueBeam accelerator, the details of the redesigned monitor chamber construction/composition/location are not available. The amount of backscatter radiation to this component and the way the chamber responds to this backscatter, therefore, cannot be directly modeled in MC. Discrepancies between the measured and MC‐simulated output factors on the TrueBeam could be a result of not taking into account the backscatter to the chamber. Ignoring the backscatter component to the chamber is equivalent to setting the value of $D_{ch}^{back}$ in Equation 8 of Popescu et al.^(43)^ to a value of zero. If this is the case, the equation for absolute dose becomes a simple linear dose conversion factor, as shown in Eq. (1):

(1) $$D_{xyz,abs}=D_{xyz}\times \frac{D_{xyz,abs}^{cal}}{D_{xyz}^{cal}}\times U$$

where $D_{xyz,abs}=\text{MC}$ absolute dose at depth (Gy); $D_{xyz}=\text{MC}$ raw dose at depth (Gy/particle‐incident‐on‐target); $D_{xyz,abs}^{cal}=\text{measured dose}/\text{MU for calibration field}*(\text{Gy}/\text{MU})$; $D_{xyz}^{cal}=\text{MC raw dose for calibration field}\;(\text{Gy}/\text{particle})$; and U=number of monitor units (MU) in plan ($*\text{calibration field}=10\times 10\;{\text{cm}}^{2}$, SAD=100 cm, d=10.0 cm).

The $D_{xyz,abs}^{cal}$ (in units of Gy/MU) is required to relate this dose to our linac absolute dose calibration field (6X beam: d=1.5 cm, $\text{field size}=10\times 10\;{\text{cm}}^{2}$). The value is equivalent to our tissue‐maximum ratio (TMR). This conversion factor technique can be validated by comparing MC calculated output factors against the measured values.

#### A.6 Postprocessing: Savitzky‐Golay smoothing filter

All clinical VMAT MC‐calculated dose distributions have a 3D locally adaptive Savitzky‐Golay smoothing filter applied. This filter fits the data from a given search window size to a polynomial using a least squares objective function.^(46)^ The use of the Savitzky‐Golay filter for denoising MC dose distributions was first reported by Kawrakow in 2002.^(47)^ The author modified the filter to provide more or less smoothing (large or small window) in an adaptive manner, based on a chi‐squared test of the least squares polynomial fit. The coefficients of the curve are selected so as to minimize the difference between the smoothed and raw data over all data points. The filter coefficients will vary as it moves across the dataset as they are influenced by both the element value and its associated uncertainty. The adaptive nature means that in low‐gradient regions, the smoothing filter will use many voxels as part of the polynomial fit. In high‐gradient regions, the smoothing window may be reduced until some data points may receive no smoothing at all. The benefit of this filter is that clinical VMAT plans can be run with two to twenty times fewer histories (i.e., two to twenty times faster simulation)^(47)^ and still achieve a final denoised distribution with a less than 1.5% uncertainty in the high‐dose region. Clinical VMAT simulations will typically use 400 million histories for the DOSXYZnrc run. The uncertainty pre‐versus post‐filtering is typically 2% vs. 1%.

### B. HD120 MLC modeling

The TrueBeam STx linac is equipped with a ‘high definition’ 120‐leaf multileaf collimator (HD120 MLC) which features two banks of 60 tungsten leaves. The central 8 cm is comprised of 32×0.25 cm wide leaves (projected at isocenter). The outer 14 cm is comprised of 28×0.50 cm wide leaves. The maximum MLC‐defined field length perpendicular to leaf motion is 22.0 cm at 100 cm from the X‐ray source. Particles are transported through the MLC using a fast, simplified model developed by Keall et al.^(33)^ and Siebers et al.,^(34)^ where only photon attenuation and single Compton scatter are accounted for. There are several configuration files that describe the geometric and compositional properties of the multileaf collimator (upperhalf.table, lowerhalf.table, particledmlc.config). The following information must be specified in these files:

A. upperhalf.table and lowerhalf.table

1. distance of upper surface of MLC region from source
2. leaf number
3. leaf thickness as a function of position (including tongue and groove geometry)

B. particlemlc.config

1. physical density of tungsten composite
2. leaf tip radius of curvature
3. leaf tip ‘tip angle’
4. maximum thickness of leaf tip
5. physical leaf offset between closed leaf pairs (MLC calibration dependent)

**Figure 1:**
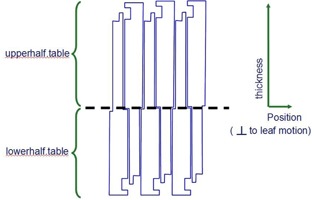
MLC configuration files describe MLC thickness as a function of position (perpendicular to leaf motion). MLC thickness is divided into two halves and described by files: upperhalf.table and lowerhalf.table.

**Figure 2:**
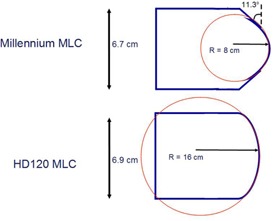
Leaf tip shape: Varian Millennium120 vs. HD120 multileaf collimators. Note: HD120 MLC does NOT have the 11.3° ‘tip‐angle’ that is part of the Millennium design. Leaf tip shape parameters in the file: particledmlc.config.

The HD120 MLC configuration files were obtained by modifying the previously validated and implemented Millennium 120 MLC configuration files. The physical descriptions of these two MLC models are available from Varian and associated references^30^, ^37^, ^48^ and are shown in Table 1 and Figs. 1 and 2.

**Table 1:** Construction differences between Varian Millennium and HD120 MLC

*Property*	*Millennium 120*	*HD120*
Leaf Width	40×0.5 cm inner	32×0.25 cm inner
	20×1.0 cm outer	28×0.50 cm outer
Max MLC‐defined Field Size(cm^2^)	40Y×40X	22Y×32X
Leaf Thickness	6.7 cm	6.9 cm
Composition	92.5% W	95% W
Density $g/{\text{cm}}^{3}$	17.15−17.85	18.0−18.53, ^(35)^ 18.7^(36)^
Leaf Tip Radius of Curvature	8 cm	16 cm
Tip Angle	11.3°	n/a

As previously reported by Lobo and Popescu,^(15)^ the particleDMLC code was also modified such that it does not call upon a particle from a phase space preceding the location of the MLC (as it was originally designed to do). Instead, DOSXYZnrc will call BEAMnrc and the particleDMLC code dynamically via the use of shared libraries to run a particle through a gantry angle‐specific aperture.

#### B.1 MLC Transmission

A MLC transmission value is calculated by obtaining ratio of closed leaf doses (one leaf bank is completely blocking the field) to open field doses across several leaves. The number of monitor units delivered to the blocked film is 4000 MU. The field size is $15\times 17\;{\text{cm}}^{2}$ and the depth of the GAFCHROMIC EBT2 (International Specialty Products, Wayne, NJ) film plane measurement in a simple solid water $40\times 40\;{\text{cm}}^{2}$ slab‐phantom geometry is 5.0 cm. Films are scanned on a Microtek Lab Inc. (Santa Fe Springs, CA) Scan Maker 9800 XL using the following settings: RGB 48 bit, 100 ppi, four‐line sampling. A mean value across several leaves (over a 12 cm span) is calculated. Percent transmission values for film measurement of the 6X beam are compared to MC simulation. The MC simulation is performed on a cube phantom with a $2\;{\text{mm}}^{3}$ voxel size. The density of the tungsten leaves in the MC MLC model is adjusted to provide a transmission value that matches the film measurement. Results are also compared to CC13 ion chamber commissioning measurements acquired in water tank.

#### B.2 Static MLC patterns

GAFCHROMIC film measured and Monte Carlo simulated absolute doses were generated for a static MLC pattern where odd‐numbered MLC leaves from one bank project into the X‐ray field to create a bar pattern. The coronal dose distributions are compared at 5.0 cm depth (SAD 100 cm) in a slab Solid Water phantom. The MC simulation is performed on a cube phantom with a $1\;{\text{mm}}^{3}$ voxel size.

#### B.3 Dynamic MLC patterns

Three dynamic MLC patterns were delivered to a GAFCHROMIC film plane in phantom (SAD 100 cm, depth=5.0 cm): 1) a multibar pattern comprising of three bar widths (7 mm, 5 mm, and 2 mm) delivered simultaneously, 2) a gradient pattern in the cross‐leaf direction, and 3) a negative pyramid pattern (gradients in both in‐ and cross‐leaf directions). The MC phantom for the 7‐5‐2 mm bar pattern is a cube with a voxel size of $1.0\;{\text{mm}}^{3}$. The voxel size for the gradient and pyramid patterns is $2.0\;{\text{mm}}^{3}$.

### C. Clinical VMAT plan verification

VMAT treatment plans using the 6X and 6X FFF beam were generated for three SABR treatment sites (SABR lung, liver, vertebral body) to test the MC model as a patient‐specific verification tool. The details of the clinical sites and treatment parameters are outlined in Table 2.

**Table 2:** Parameters used for clinical VMAT SABR examples

*Site (volume: cc)*	*Dose/Fraction (cGy)*	*Fractions*	*Field Size* $\left({\text{cm}}^{2}\right)$	*MU*
6XDose Rate=600 MU/min				
lung (17 cc)	700	5	56×5.4	2462
vertebral body (233 cc)	600	5	10.5×10.5	2142
6X FFF Dose Rate=1200 MU/min				
lung (17 cc)	700	5	5.6×5.4	4031
liver (74 cc)	1500	3	6.0×6.0	4719
vertebral body (233 cc)	600	5	10.5×10.5	2920

The patient treatment plan is transferred to two different in‐house, water‐equivalent verification phantoms. One is the 26.7 cm diameter cylindrical phantom that can accommodate a 0.6 cc volume Farmer‐type chamber (PTW‐Freiburg, Freiburg, Germany) at isocenter (SAD 100 cm).

The other phantom is the $25\times 25\times 27\;{\text{cm}}^{3}$ rounded‐corner cube phantom which can accommodate $17\times 17\;{\text{cm}}^{2}$ film sheets in one of three orthogonal directions. The film used is

GAFCHROMIC EBT2. Films are scanned on a Microtek Scan Maker 9800 XL using the following settings: RGB 48 bit, 100 ppi, four‐line sampling.

Doses are calculated to these verification phantoms using the Eclipse TPS (AAA v.10) (Varian Medical Systems) and a 1 mm calculation grid. A ‘calculated’ ion chamber dose is recorded for the cylindrical phantom by determining the mean dose to a 3D ROI representing the shape and location of the ion chamber insert. A coronal dose plane at isocenter (depth=12.5 cm) is also generated for the film phantom. Plan parameters (e.g., field size, MLC patterns, monitor units) are exported from the Eclipse TPS to the Monte Carlo cluster in DICOMRT format. The CT images for both verification phantoms were used to reconstruct a voxelized phantom ($\text{voxel size}=2.5\;{\text{mm}}^{3}$) compatible with the MC code. A command‐line script calls the necessary programs to automatically generate input files for the MC simulation and launches BEAMnrc/DOSXYZnrc. This script has been used clinically to verify over 1500 6X VMAT treatment plans on Varian iX accelerators (with Millennium 120 MLC). For the BEAMnrc and DOSXYZnrc simulations, the number of histories used in the simulation is selected to produce an uncertainty of ≤2% (typically 500 M for each simulation). MC runs use the following parameters: 1) AP=PCUT=0.010 MeV, and 2) AE=ECUT=0.700 MeV. No photon/electron splitting was invoked. Simulations are calculated on a 2005 vintage blade‐style cluster comprised of 20 Opteron (AMD, Sunnyvale, CA) 1210 1.8 GHz dual core processors (40 calculation nodes).

The DOSXYZnrc simulation generates a 3D dose matrix with associated uncertainty for each voxel. This dose matrix is denoised with the Savitzky‐Golay filter, converted to absolute dose, and written into a DICOMRT file format compatible with the Eclipse product, and then reimported back into the TPS. This step allows for a side‐by‐side plan comparison of the TPS calculated doses and the MC simulated doses. Many convenient tools for plan comparison are also available in the TPS. The TPS 3D dose matrix and the MC 3D dose matrix are compared qualitatively on screen (isodoses), then exported to MATLAB (The MathWorks, Natick, MA) where a quantitative 3D gamma factor^(49)^ is calculated. The gamma factor uses a 3% (of max TPS dose)/3 mm criteria and is applied to a ROI that includes doses that are >20% of the TPS maximum dose.

All treatment plans were delivered on the TrueBeam STx linear accelerator. The 6X plans are delivered with a maximum dose rate (dose rate is variable) of 600 MU/min. The 6XFFF plans are delivered with a maximum dose rate of 1200 MU/min. The maximum gantry speed (also variable) is $4.8^{{}^{\circ}}/s$.

## III. RESULTS

### A. Monte Carlo simulations

#### A.1 6 MV percentage depth doses/profiles

**Figure 3:**
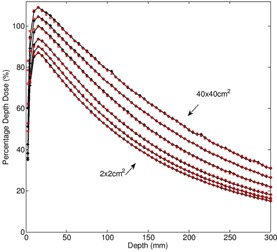
For 6X, percentage depth doses for open field sizes: $2\times 2\;{\text{cm}}^{2}$, $3\times 3\;{\text{cm}}^{2}$, $5\times 5\;{\text{cm}}^{2}$, $10\times 10\;{\text{cm}}^{2}$, $20\times 20\;{\text{cm}}^{2}$, and $40\times 40\;{\text{cm}}^{2}$. Solid line with stars=Monte Carlo,hollow squares=ion chamber measurement.

**Figure 4:**
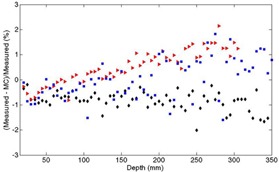
For 6X, percent difference between MC and chamber measurement along open beam PDD curve beyond $D_{\text{max}}$. $\text{Triangles}=2\times 2\;{\text{cm}}^{2}$, $\text{diamonds}=10\times 10\;{\text{cm}}^{2}$, $\text{squares}=40\times 40\;{\text{cm}}^{2}$.

**Figure 5:**
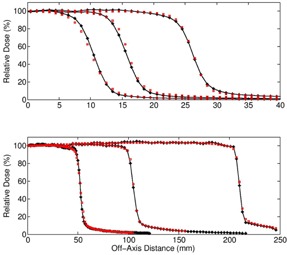
For 6X, cross‐plane, open beam profiles at a depth of 5.0 cm. Top row: field sizes are 2×2, 3×3, and 5×5. Bottom row: field sizes are 10×10, 20×20, and $40\times 40\;{\text{cm}}^{2}$. Solid line with stars=Monte Carlo,hollow squares=ion chamber measurement.

Monte Carlo simulated percentage depth dose curves for the Varian TrueBeam 6X beam are compared to measured doses, as shown in Fig. 3. Six field sizes ranging from $2\times 2\;{\text{cm}}^{3}$ to $40\times 40\;{\text{cm}}^{3}$ are shown. Doses are normalized relative to the $d_{\text{max}}$ dose for the $10\times 10\;{\text{cm}}^{2}$ field. The statistical error in the MC simulation is <1.5%. The maximum percentage dose difference between the measured and Monte Carlo curves beyond the buildup region is <2%, as illustrated in Fig. 4. The cross‐plane profiles for the same six field sizes are shown in Fig. 5.

#### A.2 Output factors

The output factor comparison between measurement and MC simulation is shown in Table 3. For all square and asymmetric field sizes listed, the agreement between measured and MC simulated output factors is ≤1.6%. For the field sizes that are clinically relevant for SABR cases ($2\times 2\;{\text{cm}}^{2}-10\times 10\;{\text{cm}}^{2}$), the agreement is within 0.9%.

**Table 3:** 6X MC simulated and measured output factor values for various fields sizes

*Field Size* $\left({\text{cm}}^{2}\right)$	*MC Output Factor (% diff from measured)*	*Measured Output Factor*
40×40	1.181 (0.0%)	1.181
20×20	1.092 (−1.0%)	1.103
10×10	‐	
4×4	0.867 (+0.2%)	0.865
3×3	0.832 (−0.1%)	0.833
2×2	0.791 (+0.9%)	0.787
40×4	0.949 (+1.6%)	0.934
4×40	0.962 (+1.0%)	0.952

### B. HD120 MLC modeling

#### B.1 MLC transmission

Transmission measurements with GAFCHROMIC film are compared to MC simulations for the 6X beam. The average transmission value (over a 12 cm span) as measured with film is 1.2%±0.1%. The transmission value calculated by MC is 1.1%±0.2%. The MC transmission data are obtained by using a physical density in the tungsten MLC model of $18.9\;g/{\text{cm}}^{3}$. The MC transmission value is consistent with that acquired during commissioning of the same unit using an ion chamber in water tank (1.2%).

#### B.2 Static MLC patterns

**Figure 6:**
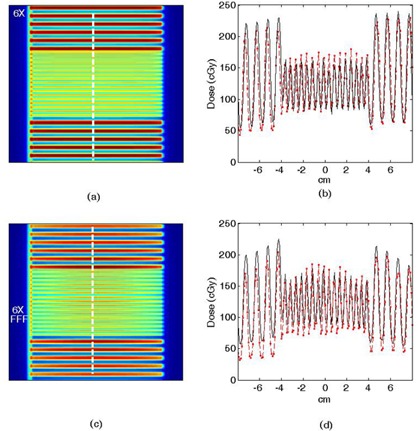
Odd numbered MLC leaves extended from Bank A: (a) 6X MC dose plane (vertical line=profile location), (b) 6X profiles, (c) 6X FFF MC dose plane, (d) 6X FFF profiles. Solid line=film, hollow squares with dashed line=MC dose.

For the static odd‐leaf projection pattern (an extreme static field test), in‐plane measured and MC dose profiles are plotted in absolute dose, as shown in Fig. 6. Note the slightly peaked dose profile due to the characteristic forward peaked intensity distribution from the 6X FFF open beam (Fig. 6(d)). There is a discrepancy between the MC and film measurement profile for the 6X FFF beam along the negative‐valued distance axis (perpendicular to leaf motion). The MC values are running low by 20%–40% across the 5.0 mm wide leaves. Potential sources of this error may be film nonuniformity or a slight (sub mm) misalignment of the two halves of the geometric MLC model (there is an upper and lower half that are stacked to create an overall MLC thickness). An increase in overall MLC thickness in this region may cause the decreased transmission observed relative to film. The source of this is still under active investigation. However, as shown in the following sections, the overall impact on the dynamic MLC verification, particularly when applied in the context of a multiangle VMAT delivery, is minimal.

#### B.3 Dynamic MLC patterns

**Figure 7:**
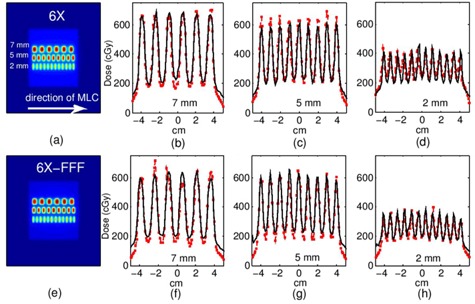
Three dynamic MLC bar patterns (7 mm, 5 mm, 2 mm) delivered simultaneously: (a) 6X dose plane, (b) 6X profiles across 7 mm bars, (c) 6X profiles across 5 mm bars, (d) 6X profiles across 2 mm bars, (e) 6X FFF dose plane, (f) 6X FFF profiles across 7 mm bars, (g) 6X FFF profiles across 5 mm bars, (h) 6X FFF profiles across 2 mm bars. Solid line=film, hollow squares=MC. Note: TPS profiles not shown for clarity.

The dynamically delivered 7‐5‐2 mm multibar pattern planar dose distributions for the 6X and 6X FFF beams are shown in Fig. 7. GAFCHROMIC film profiles are compared to MC profiles in absolute dose. Although EBT2 GAFCHROMIC film has been demonstrated to have a fairly neutral energy response,^(50)^ the film measurement curves in Fig. 7 still demonstrate some film overresponse in the field‐edge/penumbra region, compared to the MC data. This discrepancy is not present in the open‐field, cross‐plane profile comparisons between MC and ion chamber measurements, shown in Fig. 5. The film overresponse may be due to low‐dose resolution/calibration issues in this region. A similar issue was noted by other authors when comparing MC MLC dose profiles to EBT2 film measurements.^(37)^

**Figure 8:**
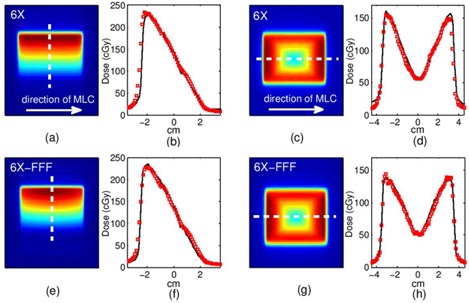
Dynamic MLC patterns. Top row=6X beam. Bottom row=6X FFF beam. Y‐gradient: (a),(e) dose planes (dashed line indicates profile position), (b),(f) profiles; Negative pyramid: (c),(g) dose planes, (d),(h) profiles. Solid line=film, hollow squares=MC.

The ‘Y’ gradient pattern delivered a ‘wedge’ shaped distribution with the 6X and 6X FFF beams, as shown in Fig. 8 (a), (b), (e), and (f). This pattern is challenging, as the gradient runs in a direction perpendicular to the dynamic leaf motion.

Finally, the negative pyramid patterns for the 6X and 6X FFF beams are shown in Fig. 8 (c), (d), (g), and (h). The dose profiles for film and MC are shown.

### C. Clinical VMAT plan verification

**Figure 9:**
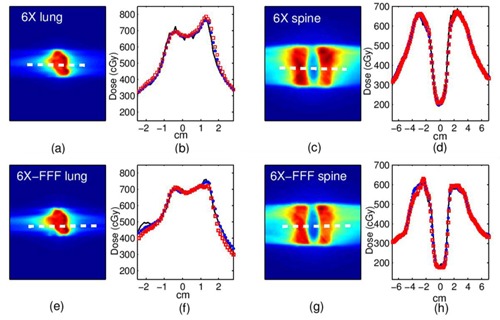
SABR clinical examples: top row=6X beam, bottom row=6X FFF beam. SABR lung: (a),(e) dose planes (dashed line indicates profile position), (b),(f) profiles; SABR vertebral body: (c),(g) dose planes, (d),(h) profiles. Solid line=film, hollow squares=MC.

The Eclipse 3D TPS dose distribution and profiles for the 6X and 6X FFF beam clinical examples are compared to Monte Carlo simulations and film measurement (see Fig. 9). The ion chamber point doses and 3D gamma factor pass rates (3%/3 mm) are shown in Table 4.

**Table 4:** 6X & 6X FFF beams: VMAT SABR patient verification results (ion chamber doses and gamma factor). 3D gamma results (%gamma values<1) are for 3%/3 mm criteria (20% ROI)

*Site (volume: cc)*	*Ion Chamber Dose (cGy)*	*TPS (% diff from meas.)*	*MC (% diff from meas.)*	*Gamma (% pass)*
6X Dose Rate=600 MU/min				
lung (17 cc)	734.3	725.8 (−1.2%)	735.5 (+0.2%)	99%
vertebral body (233 cc)	662.4	663.7 (+0.2%)	658.6 (−0.6%)	99%
6X FFF Dose Rate=1200 MU/min				
lung (17 cc)	732.6	750.4 (+2.4%)	747.5 (+2.0%)	94%
liver (74 cc)	1657.6	1658.2 (0.0%)	1668.5 (+0.7%)	98%
vertebral body (233 cc)	728.0	734.8 (+0.9%)	724.7 (−0.4%)	94%

## IV. DISCUSSION

A challenge encountered when modeling the Varian TrueBeam 6X beam is that one is limited to using a vendor‐supplied phase space located above the secondary collimator. Open field validation of 6X PDD and profile data generated using these prepackaged phase spaces indicate that they can be implemented successfully in a clinical setting. The MC‐calculated PDDs agree with measured values to within 2% for field sizes $2\times 2\;{\text{cm}}^{2}$ to $40\times 40\;{\text{cm}}^{2}$ beyond the buildup region. For the 6X FFF beams Gete et al.^(12)^ reported a maximum dose difference in the measured and MC simulated PDDs of 1%.

Initially, there was some concern with regard to not having any information about the backscatter radiation component at the level of the monitor chamber. This information is needed for other Varian linear accelerators (e.g., the iX models) to properly convert the Monte Carlo dose/particle units into absolute dose for varying field sizes (particularly for highly asymmetric field sizes).^(44)^ Ignoring the backscatter contribution to the monitor chamber may produce a systematic error in the MC simulations, particularly in the simulation of output factors.^51^, ^52^ The measured‐to‐MC comparison of output factors from the 6X beam demonstrates that it is reasonable to not account for this backscatter (maximum difference between measured and MC output factors=1.6%, even for asymmetric fields). Gete and colleagues also found that, for the 6X FFF beam, there was a <0.4% difference in measured and MC output factors for the same field sizes. This agreement may be reflective of the reduced scatter to the monitor chamber due to the missing flattening filter (for the 6X FFF case), or of a design feature of the monitor chamber rendering it less sensitive to backscatter. The absolute dose for both the 6X and 6X FFF beams can thus be calculated in a straight forward manner by applying a single conversion factor.

The HD120 MLC is modeled by adapting the particleDMLC MLC code from the original Millennium 120 leaf version.^(34)^ There are several configuration files that need to be updated to accommodate changing parameters, such as cross‐leaf thickness versus position, leaf tip radius and shape, material thickness, leaf width, composition, physical density, physical leaf offset, and MLC position offset tables (to account for rounded leaf end for different leaf positions). The physical density of $18.9\;g/{\text{cm}}^{3}$ is higher than that reported by Fix et al.^(36)^ ($18.53\;g/{\text{cm}}^{3}$) and Borges et al.^(37)^ ($18.7\;g/{\text{cm}}^{3}$). The transmission value calculated for the 6X beam (1.2%±0.1%) is consistent with ion chamber commissioning data (1.2%), as well as with that reported by the Fix (1.25%) and Borges (1.10%±0.03%) studies.

Various static and dynamic MLC patterns were used to validate the MLC model. Discrepancies in the extreme static‐field case (odd‐leaf projection test) were seen in the troughs of this bar‐like pattern, possibly due to a small misalignment between the two halves (upper and lower) of the geometric MLC model, resulting in some specific areas of variation in leaf thickness. Despite this, for simple, single‐field dynamic fields and for clinical SABR lung, vertebral body, and liver VMAT delivery, plan dose distributions were verified successfully. At the time of writing, this method has been used to validate over 140 6X and 16 6X FFF TrueBeam patient plans.

The simulations presented in this manuscript were calculated on a 2005 vintage 20x Opteron 1210 dual core processors (1.8 GHz). A total of 40 nodes are available for calculations. For clinical QA purposes, approximately eight processors per run are used (to accommodate simultaneous multipatient QA runs; in this case five patients at a time). Typically, a patient QA simulation will take 2 to 3 hours to complete. With full access to all 40 nodes, a patient VMAT run would take less than 1 hour. It should be noted that the simulations can occur during regular working hours when the physical linac is not available for QA measurements. A new MC quality assurance dedicated cluster has recently been commissioned at our clinic. For the same patient verification, the new cluster can calculate a 3D dose distribution in just under 18 minutes when using all 128 of the available cores (compared to 1 hr on the 40 vintage nodes). The new cluster is comprised of 16 Intel Xeon 2.00 GHz 8‐core CPUs (E5‐2650 0) (Intel Corporation, Santa Clara, CA) at 2.00 GHz.

The various steps of the MC VMAT simulation have been scripted such that the total person‐hours that each physicist spends on a patient MC calculation process (calculating dose to phantom in Eclipse, exporting to cluster, starting script, 3D MC dose comparison to Eclipse) is approximately 10 to 15 minutes, rendering this QA technique clinically feasible.

## V. CONCLUSIONS

The TrueBeam 6X open phase space has been independently validated for clinical use. A HD120‐leaf MLC model has been built and implemented into the Siebers‐Keall explicit‐approximate particle transport code. The new 6X and 6X FFF HD120 MLC Monte Carlo model has been validated for patient‐specific 3D verification of SABR VMAT treatment plans. This TPS 3D dose distribution validation tool has been adopted into our clinical IMRT/VMAT quality assurance program and has been used to verify over 140 6X and 16 6X FFF SABR patient plans.

## ACKNOWLEDGMENTS

The authors would like to acknowledge the Varian Monte Carlo research team for providing the phase‐space data for the 6X and 6X FFF beams. Dr. I.A. Popescu has provided some very useful discussions and insight into this topic. He also generated many of the test pattern MLC control files. Technical support from Ron Horwood, Vince Strgar, and Parmveer Atwal during the film and chamber measurement acquisitions is gratefully acknowledged.
